# YoeB toxin is activated during thermal stress

**DOI:** 10.1002/mbo3.272

**Published:** 2015-07-06

**Authors:** Brian D Janssen, Fernando Garza-Sánchez, Christopher S Hayes

**Affiliations:** 1 Department of Molecular, Cellular and Developmental Biology, University of California, Santa BarbaraSanta Barbara, California; 2 Biomolecular Science and Engineering Program, University of California, Santa BarbaraSanta Barbara, California

**Keywords:** A-site mRNA cleavage, mRNA turnover, ribosome pausing, RNase II, tmRNA

## Abstract

Type II toxin-antitoxin (TA) modules are thought to mediate stress-responses by temporarily suppressing protein synthesis while cells redirect transcription to adapt to environmental change. Here, we show that YoeB, a ribosome-dependent mRNase toxin, is activated in *Escherichia coli* cells grown at elevated temperatures. YoeB activation is dependent on Lon protease, suggesting that thermal stress promotes increased degradation of the YefM antitoxin. Though YefM is efficiently degraded in response to Lon overproduction, we find that Lon antigen levels do not increase during heat shock, indicating that another mechanism accounts for temperature-induced YefM proteolysis. These observations suggest that YefM/YoeB functions in adaptation to temperature stress. However, this response is distinct from previously described models of TA function. First, YoeB mRNase activity is maintained over several hours of culture at 42°C, indicating that thermal activation is not transient. Moreover, heat-activated YoeB does not induce growth arrest nor does it suppress global protein synthesis. In fact, *E. coli* cells proliferate more rapidly at elevated temperatures and instantaneously accelerate their growth rate in response to acute heat shock. We propose that heat-activated YoeB may serve a quality control function, facilitating the recycling of stalled translation complexes through ribosome rescue pathways.

## Introduction

In *Escherichia coli*, prolonged translational arrest often leads to mRNA degradation into the ribosome A site (Hayes and Sauer 2003; Sunohara et al. 2004; Li et al. 2006, 2008; Garza-Sánchez et al. 2008). This A-site mRNA cleavage activity results in truncated A-site codons, which prevent further decoding and produce stalled translation complexes. Such non-productive ribosomes are “rescued” by at least three quality control systems in bacteria (Janssen and Hayes 2012). tmRNA-SmpB mediates the primary ribosome rescue pathway, and this system is found in all eubacteria and some plastids (Tu et al. 1995; Keiler et al. 1996, 2000; Gueneau de Novoa and Williams 2004). tmRNA is a stable RNA with both transfer-RNA and messenger-RNA functions and recycles stalled ribosomes in a process termed *trans-*translation. During *trans*-translation, tmRNA-SmpB enters the ribosome A site, and the nascent peptide is transferred to alanine-charged tmRNA. The truncated message is then released from the ribosome and translation resumes using a short open reading frame within tmRNA. In this manner, tmRNA provides a stop codon in *trans*, thereby allowing normal translation termination and ribosome recycling (Hayes and Keiler 2010). More recently, two alternative ribosome rescue pathways have been identified. ArfA (alternative rescue factor A) was discovered in a synthetic lethal screen for genes that are essential for the viability of mutants that lack tmRNA (Chadani et al. 2010). ArfA is a small peptide that binds the ribosome and allows release factor-2 to catalyze nascent chain release in the absence of an A-site stop codon (Chadani et al. 2012; Shimizu 2012). YaeJ (ArfB) is a release factor homolog that lacks the canonical stop codon recognition domain (Baranov et al. 2006; Hayes and Keiler 2010). Thus, YaeJ can bind the ribosome A site in the absence of a stop codon and catalyze nascent peptide release (Chadani et al. 2011; Handa et al. 2011; Gagnon et al. 2012; Feaga et al. 2014). Because A-site cleavage produces nonstop transcripts in response to translational pausing, this nuclease activity is thought to function in molecular quality control by facilitating ribosome rescue (Hayes and Sauer 2003; Sunohara et al. 2004).

In *E. coli* there are at least two enzymes, RelE and YoeB, which have ribosome-dependent A-site nuclease activity (Pedersen et al. 2003; Feng et al. 2013). RelE and YoeB are encoded by type II toxin–antitoxin (TA) modules together with cognate antitoxins that specifically neutralize nuclease activity. These mRNases have been termed “toxins” because their activities inhibit cell growth and can lead to cell death under some circumstances. In general, toxins are activated under stress or other conditions that prevent continued antitoxin synthesis. Antitoxins are labile to proteolysis and their degradation liberates the cognate toxins to exert growth inhibition activities. The physiological functions of TA systems remain controversial. They have been proposed to play roles in stress-response, persistence, genomic stability and programmed cell death (Engelberg-Kulka et al. 2005; Gerdes et al. 2005; Magnuson 2007; Tsilibaris et al. 2007; Nariya and Inouye 2008; Maisonneuve et al. 2011). Though RelE and YoeB have the potential to catalyze A-site mRNA cleavage during translational arrest, mutants lacking these enzymes and other known toxins retain A-site cleavage activity (Hayes and Sauer 2003; Sunohara et al. 2004; Garza-Sánchez et al. 2009; Janssen et al. 2013). Thus, the identity of the A-site nuclease is unknown, but it remains possible that an unidentified toxin catalyzes cleavage in response to the stress of translational pausing.

Though the A-site nuclease has not been identified, the phenomenon clearly requires the 3′-to-5′ exoribonuclease activity of RNase II (Garza-Sánchez et al. 2009; Janssen et al. 2013). In Δ*rnb* mutants, which lack RNase II, prolonged translational arrest produces transcripts that are truncated 12 nucleotides downstream of the ribosome A site (Garza-Sánchez et al. 2009; Janssen et al. 2013). This position corresponds to the “toeprint” of the paused ribosome, suggesting that the ribosome physically blocks further mRNA degradation into the A site. However, purified RNase II only degrades mRNA to the +18 position (with respect to the A-site codon) when incubated with translation complexes in vitro (Garza-Sánchez et al. 2009). Moreover, the deeply recessed active site of RNase II is incompatible with the ability to directly catalyze A-site mRNA cleavage (Frazao et al. 2006; Zuo et al. 2006). Because RNase II is unable to degrade the A-site codon itself, the enzyme must play an indirect role in A-site mRNA cleavage. One possibility is that RNase II-mediated mRNA degradation to the 3′-edge of the ribosome is required for subsequent degradation into the A site by an unknown nuclease. This model is supported by experiments showing that secondary structures placed on the 3′-side of paused ribosomes are sufficient to inhibit RNase II activity and also block A-site cleavage (Garza-Sánchez et al. 2009).

We previously reported that A-site mRNA cleavage is suppressed in response to heat shock at 42°C (Garza-Sánchez et al. 2009). This observation suggests that A-site nuclease activity is heat labile, and therefore we revisited these experiments in an effort to identify the enzyme responsible. Although acute heat shock temporarily inhibits A-site mRNA cleavage, the experiments presented herein demonstrate that prolonged growth at elevated temperature actually restores the activity. This heat-induced mRNase activity is dependent on the Lon protease, suggesting the activation of one or more TA modules during thermal stress. Taking a candidate-gene approach, we discovered that the *yefM-yoeB* TA operon is required for heat-induced A-site mRNA cleavage activity. Remarkably, YoeB mRNase activity remains constant over several hours of culture at 42°C. Cells proliferate rapidly under these conditions, demonstrating that growth arrest is not necessarily concomitant with toxin activation. Moreover, global protein synthesis is not suppressed by the active toxin. Together, these findings show that type II toxins can be activated at low levels in response to environmental stress. We propose that YoeB facilitates the recycling of stalled translation complexes, thereby playing a role in molecular quality control during thermal stress.

## Materials and Methods

### Bacterial strains and growth conditions

All bacterial strains were derived from *E. coli* X90 (DE3) and are listed in Table1. The Δ*clpB::kan*, Δ*htpG::kan*, Δ*lon::kan,* and Δ*clpP::kan* alleles were obtained from the Keio collection (Baba et al. 2006) and were transferred between strains using bacteriophage P1-mediated transduction (Moore 2011). The Δ*dnaK::kan* disruption was generated as described (Hayes and Sauer 2003). Briefly, a region upstream of *dnaK* was amplified with primers (restriction sites are underlined) dnaK-Sac (5′-GAT GAG CTC CCA CTA GTT TAC TGC TGA TAA AGA G) and dnaK-Bam (5′-AAC GGA TCC ACT ATA TAT TCG GTC ATC ATG TGG); and a downstream region with dnaK-Eco (5′-GCT GAA TTC GAA GAA GTC AAA GAC AAA AAA TAA TCG) and dnaK-Kpn (5′-AAC GGT ACC AAA AAT ATC GCT GAA GTC TGC GCC). The two polymerase chain reaction (PCR) products were sequentially ligated to plasmid pKAN to generate pKAN-dnaK. Plasmid pKAN-dnaK was digested with SacI/KpnI and the small fragment was used to delete the *dnaK* gene by Red-mediated recombination (Thomason et al. 2014). Kanamycin-resistant cassettes were removed with flippase (FLP) recombinase to allow the construction of strains carrying multiple gene deletions (Cherepanov and Wackernagel 1995). All mutations were confirmed by locus-specific PCR amplification.

**Table 1:** Bacterial strains and plasmids

Strain or plasmid	Description	Reference
Strains		
X90	F*′ lacI*^*q*^ *lac′ pro′/ara Δ[lac-pro] nalA argE[am] rif*^*r*^ *thi-1*	Beckwith and Signer (1966)
CH12	X90 (DE3)	Hayes et al. (2002a)
CH113	X90 (DE3) *ssrA::cat*, Cm^R^	Hayes et al. (2002a)
CH165	X90 *ΔssrA*	This study
CH950	X90 (DE3) *Δlon::kan*, Kan^R^	This study
CH951	X90 (DE3) *ssrA::cat Δlon::kan*, Cm^R^ Kan^R^	This study
CH972	X90 (DE3) *ssrA::cat ΔrelBE::kan*, Cm^R^ Kan^R^	Hayes and Sauer (2003)
CH1019	X90 (DE3) *ssrA::cat ΔyefM-yoeB::kan*, Cm^R^ Kan^R^	Hayes and Sauer (2003)
CH1023	X90 (DE3) *ssrA::cat ΔdinJ-yafQ::kan*, Cm^R^ Kan^R^	Hayes and Sauer (2003)
CH1129	X90 *ΔyefM-yoeB::kan*, Kan^R^	Hayes and Sauer (2003)
CH1207	X90 (DE3) *ssrA::cat Δrnb::kan*, Cm^R^ Kan^R^	Garza-Sánchez et al. (2009)
CH3550	X90 (DE3) *ssrA::cat ΔrelBE ΔchpBIK ΔyefM-yoeB ΔmazEF ΔdinJ-yafQ ΔyhaV*, Cm^R^	Garza-Sánchez et al. (2009)
CH4646	X90 *Δrnb ssrA::cat*, Cm^R^	This study
CH5820	X90 *Δrnb*	This study
CH6157	X90 (DE3) *ΔssrA ΔclpPX-lon::cat*, Cm^R^	This study
CH6158	X90 (DE3) *ΔssrA ΔclpPX-lon::cat Δrnb::kan*, Cm^R^ Kan^R^	This study
CH6262	X90 (DE3) *ssrA::cat ΔclpP::kan*, Cm^R^ Kan^R^	This study
CH6595	X90 (DE3) *ssrA::cat ΔrelBE ΔchpBIK ΔyefM-yoeB ΔmazEF ΔdinJ-yafQ ΔyhaV Δrnb::kan*, Cm^R^ Kan^R^	This study
CH6608	X90 (DE3) *ssrA::cat Δrnb ΔclpP::kan*, Cm^R^ Kan^R^	This study
CH6609	X90 (DE3) *ssrA::cat Δrnb Δlon::kan*, Cm^R^ Kan^R^	This study
CH7212	X90 (DE3) *ssrA::cat Δrnb ΔrelBE::kan*, Cm^R^ Kan^R^	This study
CH7213	X90 (DE3) *ssrA::cat Δrnb ΔyefM-yoeB::kan*, Cm^R^ Kan^R^	This study
CH7214	X90 (DE3) *ssrA::cat Δrnb ΔdinJ-yafQ::kan*, Cm^R^ Kan^R^	This study
CH7215	X90 (DE3) *ssrA::cat ΔmazEF::kan*, Cm^R^ Kan^R^	Hayes and Sauer (2003)
CH7216	X90 (DE3) *ssrA::cat Δrnb ΔmazEF::kan*, Cm^R^ Kan^R^	This study
CH7217	X90 (DE3) *ssrA::cat ΔchpBIK::kan*, Cm^R^ Kan^R^	Hayes and Sauer (2003)
CH7218	X90 (DE3) *ssrA::cat Δrnb ΔchpBIK::kan*, Cm^R^ Kan^R^	This study
CH7219	X90 (DE3) *ssrA::cat ΔyhaV::kan*, Cm^R^ Kan^R^	This study
CH7220	X90 (DE3) *ssrA::cat Δrnb ΔyhaV::kan*, Cm^R^ Kan^R^	This study
CH7360	X90 *ΔssrA ΔyefM-yoeB::kan*, Kan^R^	This study
CH7361	X90 *ΔssrA Δlon::kan*, Kan^R^	This study
CH7362	X90 (DE3) *ΔyefM-yoeB::kan*, Kan^R^	This study
CH7440	X90 *Δrnb ssrA::cat ΔyefM-yoeB::kan,* Cm^R^ Kan^R^	This study
CH7442	X90 *Δrnb ssrA::cat Δlon::kan*, Cm^R^ Kan^R^	This study
CH12151	X90 (DE3) *ssrA::cat Δrnb ΔdnaK::kan*, Cm^R^ Kan^R^	This study
CH12301	X90 (DE3) *ssrA::cat Δrnb ΔhtpG::kan*, Cm^R^ Kan^R^	This study
CH12314	X90 (DE3) *ssrA::cat Δrnb ΔclpB::kan*, Cm^R^ Kan^R^	This study
CH12402	X90 (DE3) *ssrA::cat Δrnb ΔyefM-yoeB ΔdnaK::kan*, Cm^R^ Kan^R^	This study
CH12403	X90 (DE3) *ssrA::cat Δrnb Δlon* Δ*dnaK::kan*, Cm^R^ Kan^R^	This study
Plasmids		
pSIM6	Temperature-induced expression of phage *λ* Red recombinase proteins, Amp^R^	Datta et al. (2006)
pCP20	Temperature-induced expression of FLP recombinase, Amp^R^ Cm^R^	Cherepanov and Wackernagel (1995)
pCH450	pACY184 derivative carrying arabinose-inducible *araBAD* promoter and *araC*, Tet^R^	Hayes and Sauer (2003)
pKAN	pBluescript SK+ with FRT-flanked kanamycin-resistant cassette, Amp^R^, Kan^R^	Hayes et al. (2002a)
pKAN-dnaK	Construct for deletion of *Escherichia coli dnaK*, Amp^R^ Kan^R^	This study
pFLAG-(m)YbeL-PP	Expresses FLAG epitope fused to the C-terminal 49 residues of YbeL(E159P), Amp^R^	Janssen and Hayes (2009)
pFLAG-(m)YbeL(E28Am)-PP	Variant of FLAG-(m)YbeL-PP with amber termination codon at Glu28, Amp^R^	This study
pCH450-*lon*	Arabinose-inducible expression of *lon*, Tet^R^	This study
pCH450-*lon(S679A)*	Arabinose-inducible expression of catalytically inactive *lon*, Tet^R^	This study
pCH450-*yoeB*	Arabinose-inducible expression of *yoeB*, Tet^R^	This study
pCH450-*yefM*	Arabinose-inducible expression of *yefM*, Tet^R^	This study
pCH450-*yefM-yoeB*	Arabinose-inducible expression of *yefM-yoeB* operon, Tet^R^	This study
pCH410-*relB*	Arabinose-inducible expression of *relB*, Tet^R^	This study
pCH450-*rpoH*	Arabinose-inducible expression of *σ*^32^ heat-shock transcription factor, Tet^R^	This study

Amp^R^, ampicillin resistant; Cm^R^, chloramphenicol resistant; Kan^R^, kanamycin resistant; Tet^R^, tetracycline resistant.

*Escherichia coli* cells were grown in lysogeny broth (LB) media supplemented with the appropriate antibiotics (ampicillin, 150 *μ*g/mL; chloramphenicol, 66 *μ*g/mL; kanamycin, 50 *μ*g/mL; and tetracycline 25 *μ*g/mL). Cells from overnight cultures were resuspended at optical density (OD_600_) ∼0.05 in fresh LB supplemented with the appropriate antibiotics. Cells were grown to mid-log phase (at the indicated temperatures) with shaking, then *flag-(m)ybeL-PP* expression was induced with isopropyl *β*-D-1-thiogalactopyranoside (IPTG) at a final concentration of 1.5 mmol/L. After 30 min, the induced cultures were poured into an equal volume of ice-cold methanol to arrest growth. The effects of Lon, YefM, YoeB, RelB, and *σ*^32^ on mRNA processing were determined by expressing the corresponding genes from a plasmid-borne P_BAD_ promoter (Hayes and Sauer 2003). Cells were grown to mid-log phase at the indicated temperatures and *flag-(m)ybeL-PP* induced with 1.5 mmol/L IPTG. After 30 min, l-arabinose was added to 0.4% and incubation continued for an additional 15 min. Cultures were then poured into an equal volume of ice-cold methanol. Cells were harvested by centrifugation and the cell pellets frozen at −80°C for subsequent RNA isolation. The growth-rate response to acute heat shock was determined in LB medium without antibiotics. Cells were grown for 2.5 h at 30°C in an environmental shaker. Cultures were then split in two, with one culture maintained at 30°C while the other was transferred to a shaking-water bath equilibrated at 42°C. The growth of each culture was monitored during the acute heat shock and for an additional 4.5 h. Long-term growth at various temperatures was performed on LB agar without antibiotics. Cells were grown to mid-log phase in shaking LB medium at 30°C. Culture density was adjusted to OD_600_ = 1.0, then subjected to 10-fold serial dilutions in LB medium. Samples (2 *μ*L) from each dilution were spotted onto LB agar and incubated at the indicated temperatures for 15 h.

### Plasmid constructs

Plasmid pFLAG-(m)YbeL-PP has been described previously (Janssen and Hayes 2009; Seidman et al. 2011). An amber stop codon was introduced at codon 28 of pFLAG-(m)YbeL-PP using the megaprimer PCR approach (Aiyar and Leis 1993). Plasmid pFLAG-(m)-YbeL-PP was first amplified with primers Glu28Amb (5′-GGC TGG GAA ATC TGG TCT GCT AGA AAT GTC ACT TCC ATC TCC) and pET-Eco (5′-CGT CTT CAA GAA TTC TCA TGT TTG ACA GC). The resulting product was used as a megaprimer with pET-Sph/Pst (5′-CAA GGA ATG GTG CAT GCC TGC AGA TGG CGC CC) to amplify the *flag-(m)ybeL-PP* coding sequence and T7 promoter. The final product was digested with SphI/EcoRI and ligated to plasmid pET11d. The *relB, rpoH, yefM, and yoeB* genes were all amplified from *E. coli* genomic DNA and ligated to plasmids pCH410 or pCH450 to generate l-arabinose-inducible expression constructs. The *relB* gene was amplified with relB-Nde (5′ - GAG GTG TAA CAT ATG GGT AGC ATT AAC CTG CG) and relB-Sac-rev (5′-AAT GAG CTC TCA GAG TTC ATC CAG CGT CAC ACG), digested with NdeI/SacI and ligated to plasmid pCH410 (Hayes and Sauer 2003). The *rpoH* gene was amplified with rpoH-Eco (5′-ATA GAA TTC AAG GAG ATA TCA TAT GAC TGA CAA AAT GCA AAG TTT AGC) and rpoH-Xho (5′-TAT CTC GAG AAA TTA CGC TTC AAT GGC AGC), digested with EcoRI/XhoI and ligated to plasmid pCH450. The *yefM* gene was amplified with yefM-Eco (5′-TTT GAA TTC CAT ATG AAC TGT ACA AAA GAG G) and yefM-Sac (5′-TGA GAG CTC AGA CCA GAT TAG TTT CAC TCA ATG ATG), digested with EcoRI/SacI and ligated to pCH450. The *yoeB* gene was amplified with yoeB-Eco (5′-GGA GAA TTC CAT ATG AAA CTA ATC TGG TCT GAG G) and yoeB-Sac (5′-ATA GAG CTC CGC TAG CGT ATC AAA ACT GAC AAT TC), digested with EcoRI/SacI and ligated to pCH450. The *yefM-yoeB* operon was amplified with primers yefM-Eco and yoeB-Sac, digested with EcoRI/SacI and ligated to pCH450. The wild-type and Ser679Ala alleles of *lon* were excised from plasmids pBAD33::*lon* and pBAD33::*lon(S679A)* (Gur and Sauer 2008) by SacI/SbfI digestion, and the fragments ligated to SacI/PstI-digested plasmid pCH450.

### RNA isolation and analysis

Total RNA was isolated from frozen *E. coli* cell pellets using guanidinium isothiocyanate-phenol extraction as described (Garza-Sánchez et al. 2006). RNA was quantified by absorbance at 260 nm and 10 *μ*g run on Tris-borate-ethylenediamine tetraacetic acid, 10% polyacrylamide gels containing 50% urea. Gels were electroblotted onto Nytran Supercharge nylon membranes and subjected to northern blot hybridization using oligonucleotide probes as described (Hayes and Sauer 2003; Garza-Sánchez et al. 2006). Radiolabeled oligonucleotide T7-SD probe (5′-GTA TAT CTC CTT CTT AAA GTT AAA C) was used as a probe to detect *flag-(m)ybeL-PP* transcript. Endogenous transcripts were detected with radiolabeled oligonucleotides: *lpp* 5′-probe (5′-CAT TAT TAA TAC CCT CTA GAT TGA G); *lpp* 3′-probe (5′-CTT GCG GTA TTT AGT AGC CAT G); *ompA* 5′-probe (5′-CAT TTT TTG CGC CTC GTT ATC ATC); *grpE* 5′-probe (5′-CAT GAA TTT CTC CGC GTT TTT TTC G); and *ibpB* 5′-probe (5′-CAT AGT CAT TTC TCC TTC TAA GAA GC). All northern blots were imaged with an FX phosphorimager using the Quantity One software package (Bio-Rad, Hercules, CA USA).

### Immunoblot analysis

Protein was extracted from frozen cells using freeze-thaw in urea lysis buffer (50% urea, 10 mmol/L Tris-HCl [pH 8.0], 150 mmol/L NaCl) and lysates clarified by centrifugation at 15,000 × g for 15 min (Hayes et al. 2002b). Protein was quantified by the Bradford method and equal amounts of total urea-soluble protein were run on Tris–tricine sodium dodecyl sulfate (SDS) 10% polyacrylamide gels. Immunoblot analysis was performed as described (Janssen and Hayes 2009), and blots were imaged using a Odyssey® infrared imager (LI-COR, Lincoln, NE, USA). Lon antigen was detected with rabbit polyclonal antisera and IRDye® 680 (LI-COR, Lincoln, NE, USA) labeled anti-rabbit secondary antibodies.

## Results

### A-site nuclease activity is induced at elevated temperature

**Figure 1:**
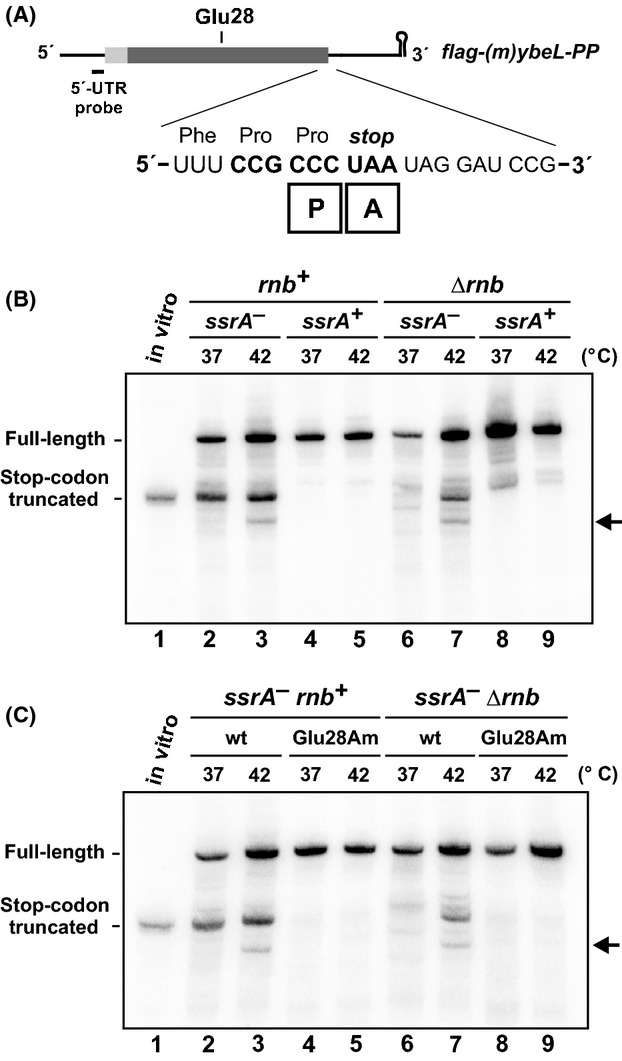
A-site nuclease activity is induced at elevated temperature. (A) The *flag-(m)ybeL-PP* reporter transcript is presented schematically. The sequence expansion depicts the P- and A-site codons during translation termination, and the position of the glutamate-28 codon is indicated. The 5′-UTR northern blot probe hybridizes immediately upstream of the start codon. (B) Northern blot analysis of A-site mRNA cleavage. *flag-(m)ybeL-PP* transcripts were expressed in cells of the indicated genotype at 37°C and 42°C, and total RNA was analyzed by northern hybridization. (C) Northern blot analysis of *flag-(m)ybeL-PP* transcripts carrying the Glu28 amber mutation (Glu28Am). Wild-type (wt) and Glu28Am transcripts were expressed in the indicated genetic backgrounds at 37°C and 42°C and analyzed by northern hybridization. The migration positions of stop codon truncated messages in (B and C) are indicated by control transcripts prepared by in vitro transcription. The horizontal arrows in (B and C) indicate an additional truncated transcript that is produced during growth at 42°C.

We previously reported that A-site mRNA cleavage is suppressed during heat shock (Garza-Sánchez et al. 2009), suggesting that the A-site nuclease is thermolabile. Alternatively, abrupt increases in temperature could disrupt protein synthesis temporarily and therefore indirectly affect A-site cleavage. To test this possibility, we revisited these experiments using the previously described *flag-(m)ybeL-PP* transcript as a reporter of A-site mRNA cleavage (Fig.1A) (Garza-Sánchez et al. 2009; Janssen and Hayes 2009). This transcript encodes a C-terminal Pro–Pro peptide motif, which interferes with translation termination and induces cleavage at the A-site stop codon (Mottagui-Tabar et al. 1994; Bjornsson et al. 1996; Hayes et al. 2002a; Hayes and Sauer 2003; Garza-Sánchez et al. 2008). A substantial proportion of *flag-(m)ybeL-PP* transcripts is truncated in the stop codon when expressed in *ssrA*^*–*^ mutants, which lack tmRNA, but not in *ssrA*^+^ cells (Fig.1B, compare lanes 2 and 4). Presumably, mRNA processing also occurs in wild-type cells, but the truncated messages are rapidly degraded once released from the stalled ribosome through tmRNA activity (Hayes and Sauer 2003; Yamamoto et al. 2003). As reported (Garza-Sánchez et al. 2009), RNase II (encoded by the *rnb* gene) is required for this activity because truncated transcripts were not detected in *ssrA*^*−*^ Δ*rnb* cells (Fig.1B, lane 6). We next examined RNA isolated from cells that had been grown at 42°C for 2 h, and unexpectedly found that A-site mRNA cleavage was not suppressed (Fig.1B, lane 3). In fact, growth at 42°C actually restored A-site cleavage activity to *ssrA*^*−*^ *Δrnb* cells (Fig.1B, compare lanes 6 and 7). Another smaller truncated transcript was also detected in *ssrA*^*−*^ cells grown at 42°C (Fig.1B, marked by an arrow in lanes 3 and 7). These observations suggest that a new RNase activity is induced during growth at 42°C. Because tmRNA promotes nonstop mRNA turnover during ribosome rescue (Yamamoto et al. 2003; Richards et al. 2006; Ge et al. 2010), the apparent absence of truncated mRNA in *ssrA*^*+*^ cells suggests that cleavage may occur during translation. We tested this hypothesis by introducing an amber stop codon at position Glu28 in the *flag-(m)ybeL-PP* coding sequence (Fig.1A). Thus, ribosomes terminate translation at codon 28 in the Glu28Am transcript, rather than the original ochre stop at codon 61. Truncated Glu28Am transcripts were not detected in any of the examined backgrounds (Fig.1C), indicating that translation to the tandem Pro codons is required for the heat-induced mRNase activity.

### YoeB mediates A-site mRNA cleavage at elevated temperature

**Figure 2:**
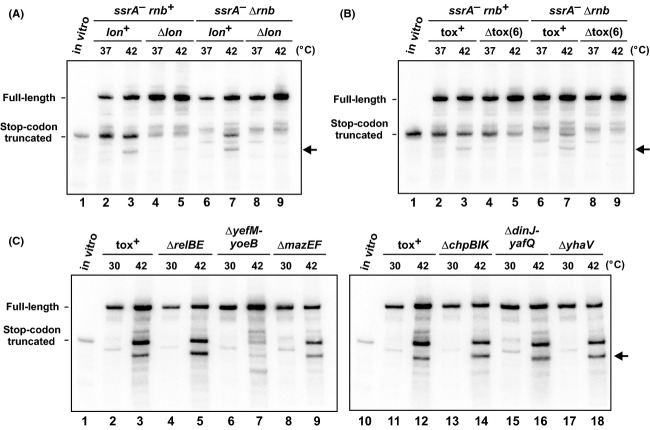
Lon and YoeB are required for temperature-induced A-site mRNA cleavage. (A) *flag-(m)ybeL-PP* transcripts were expressed in the indicated genetic backgrounds at 37°C and 42°C and analyzed by northern hybridization. (B) *flag-(m)ybeL-PP* transcripts were expressed in cells that lack six characterized toxin-antitoxin modules (Δtox(6)) at 37°C and 42°C, and compared to background that retain these toxin-antitoxin genes (tox^+^). (C) *flag-(m)ybeL-PP* transcripts were expressed in *ssrA*^*−*^ *Δrnb* cells that carry deletions in the indicated toxin–antitoxin genes. Growth at 42°C induces truncated mRNA in all cells except those deleted for *yefM-yoeB*. The migration positions of stop codon truncated messages are indicated by control transcripts prepared by in vitro transcription. The horizontal arrows indicate an additional truncated transcript that is produced during growth at 42°C.

Some type II TA modules encode ribosome-dependent RNases that cleave A-site codons (Pedersen et al. 2003; Prysak et al. 2009; Feng et al. 2013). Toxins are typically activated through Lon protease-mediated degradation of antitoxins (Gerdes and Maisonneuve 2012; Brzozowska and Zielenkiewicz 2013); therefore we examined *flag-(m)ybeL-PP* transcript processing in Δ*lon* cells. We observed less truncated mRNA in Δ*lon* compared to *lon*^*+*^ backgrounds even at 37°C (Fig.2A, lanes 2 and 4), indicating that Lon influences A-site cleavage during translational pauses. But more importantly, we failed to detect heat-induced mRNase activity in Δ*lon* mutants (Fig.2A, lanes 7 and 9). The latter result suggests that a toxin is responsible for heat-induced mRNase activity, which led us to test an *E. coli* strain that lacks multiple TA genes. The *E. coli* Δtox(6) strain lacks six validated TA systems encoded by the *relBE, mazEF, yefM-yoeB, dinJ-yafQ, chpBIK* and *yhaV* genes. As reported previously (Garza-Sánchez et al. 2009), *flag-(m)ybeL-PP* transcripts still undergo A-site cleavage in the *ssrA*^*−*^ *Δ*tox(6) background at 37°C (Fig.2B, lanes 2 and 4). However, *ssrA*^*−*^ *Δ*tox(6) *Δrnb* cells did not exhibit heat-induced mRNase activity (Fig.2B, lanes 7 and 9), strongly suggesting that one (or more) of the deleted toxins is responsible for activity. Further analysis of *ssrA*^*−*^ *Δrnb* strains carrying individual TA gene deletions revealed that *ΔyefM-yoeB* mutants lack the heat-induced activity (Fig.2C).

**Figure 3:**
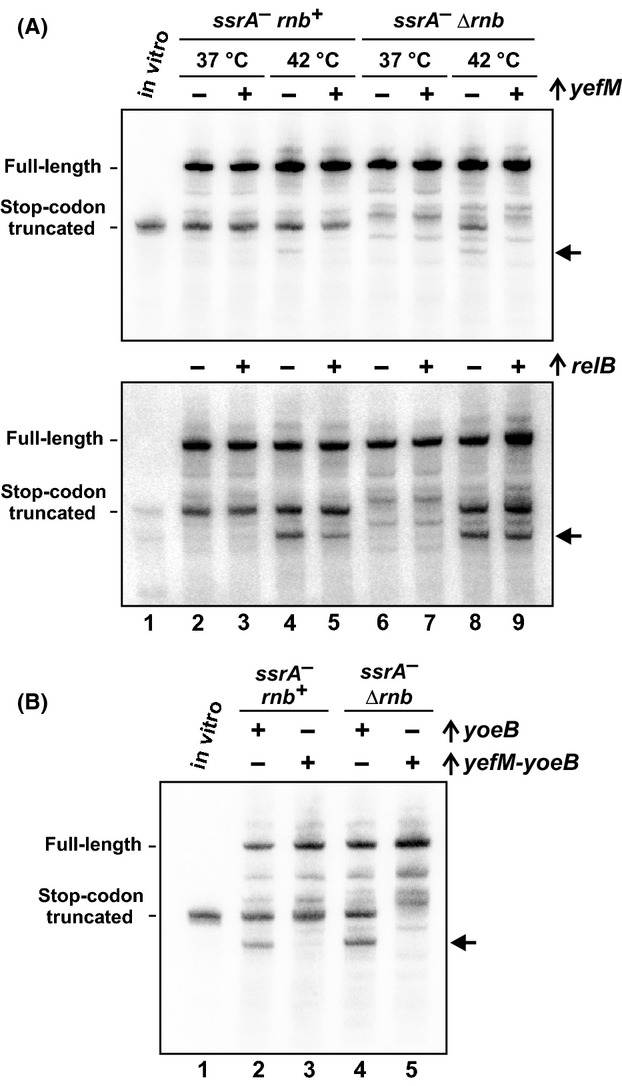
Overexpression of *yefM* suppresses temperature-induced A-site mRNA cleavage. (A) *flag-(m)ybeL-PP* transcripts were expressed in *ssrA*^*−*^ *rnb*^*+*^ and *ssrA*^*−*^ *Δrnb* backgrounds at 37°C and 42°C. Where indicated (+), the *yefM* or *relB* antitoxin genes were overexpressed from a plasmid-borne arabinose-inducible promoter. (B) *flag-(m)ybeL-PP* transcripts were expressed in *ssrA*^*−*^ *rnb*^*+*^ and *ssrA*^*−*^ *Δrnb* backgrounds at 37°C. Where indicated (+), the *yoeB* or *yefM-yoeB* genes were overexpressed from plasmid-borne arabinose-inducible promoters. The migration positions of stop codon truncated messages are indicated by control transcripts prepared by in vitro transcription. The horizontal arrows indicate an additional truncated transcript that is produced during growth at 42°C (A) or *yoeB* induction without *yefM* (B).

The YefM antitoxin specifically binds to YoeB toxin and neutralizes its RNase activity (Cherny et al. 2005; Kamada and Hanaoka 2005; Feng et al. 2013). Therefore, if heat-induced transcript cleavage is mediated by YoeB, then the activity should be specifically blocked by *yefM* overexpression. We cloned *yefM* under control of the P_BAD_ promoter and induced expression in cells that coexpress *flag-(m)ybeL-PP*. In cells grown at 37°C, *yefM* expression had no discernible effect on mRNA cleavage (Fig.3A, lanes 2, 3, 6, and 7). However, *yefM* expression suppressed mRNA cleavage in *ssrA*^*−*^ *Δrnb* cells at 42°C (Fig.3A, lane 8 and 9). This suppressive effect was specific because induction of *relB*, which encodes the antitoxin for RelE toxin, had little effect on heat-induced mRNase activity (Fig.3A, bottom blot). We then expressed *yoeB* from a plasmid-borne P_BAD_ promoter to determine whether the toxin cleaves *flag-(m)ybeL-PP* transcripts. The two major truncated species that accumulate during thermal stress were also produced in response to *yoeB* induction at 37°C (Fig.3B, lanes 2 and 4). Thus, the heat-induced mRNA activity can be recapitulated by *yoeB* expression at lower temperature. In contrast, expression of the entire *yefM-yoeB* operon from the same plasmid vector did not induce mRNA cleavage (Fig.3B, lanes 3 and 5). Taken together, these data indicate that YoeB is responsible for the heat-induced mRNase activity.

### YoeB is activated during heat-shock stress

**Figure 4:**
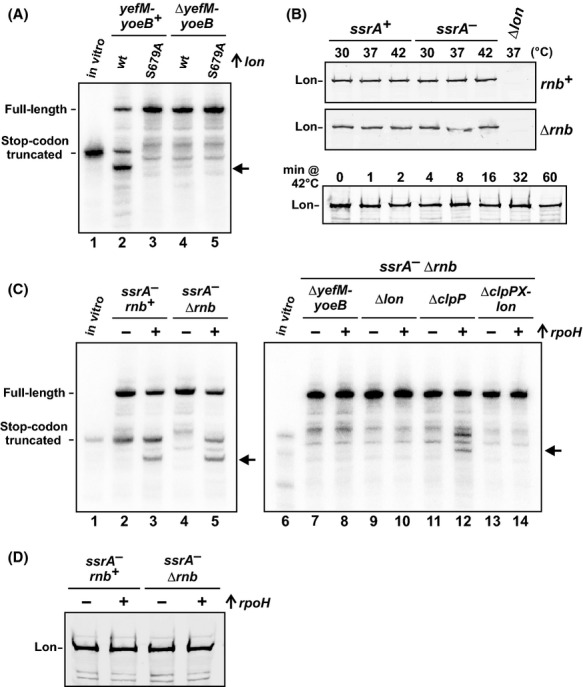
Overexpression of *lon* and *rpoH* induces A-site mRNA cleavage. (A) Overexpression of *lon* induces A-site mRNA cleavage. *flag-(m)ybeL-PP* transcripts were expressed in *ssrA*^*−*^ *Δrnb* backgrounds at 37°C. Where indicated, the *lon* or *lon(S679A)* genes were overexpressed from a plasmid-borne arabinose-inducible promoter. (B) Lon immunoblot analysis. Urea-soluble protein was isolated from cells of the indicated genotype that had been cultures at 30°C, 37°C or 42°C for 2.5 h. The bottom panel shows Lon levels in *ssrA*^*−*^ *Δrnb* cells that had been cultured at 30°C for 1.5 h, then shifted to 42°C for the indicated number of minutes. (C) Overexpression of *rpoH* induces A-site mRNA cleavage. *flag-(m)ybeL-PP* transcripts were expressed in the indicated genetic backgrounds at 37°C. Where indicated (+), *rpoH* was overexpressed from a plasmid-borne arabinose-inducible promoter. In (A and C), the migration position of *flag-(m)ybeL-PP* transcript that is truncated at the stop codon is indicated, and horizontal arrows indicate an additional *yoeB*-dependent transcript. (D) Immunoblot analysis of Lon. Urea-soluble protein was isolated from cells of the indicated genotype that had been grown at 37°C. Where indicated (+), the *σ*^32^ heat-shock transcription factor (*rpoH*) was overexpressed. Samples were analyzed by immunoblot using polyclonal antisera to Lon protease.

Christensen et al. 2004 have shown that overproduced Lon inhibits cell growth largely by activating YoeB. These findings indicate that YefM is particularly susceptible to proteolysis and suggest that thermal activation may be due to increased Lon levels. To address this possibility, we first confirmed that truncated *flag-(m)ybeL-PP* transcripts accumulate in cells that overexpress *lon* at 37°C (Fig.4A, lane 2). This mRNA processing was not observed when *lon* was induced in Δ*yefM-yoeB* cells (Fig.4A, lane 4). We also determined that protease activity is required for this effect, because truncated messages were not detected when catalytically inactive Lon(Ser679Ala) was overproduced (Fig.4A, lane 3) (Botos et al. 2004). Having established that increased Lon is sufficient to activate YoeB in our system, we then examined endogenous Lon levels by immunoblot. Given that Lon is considered to be a heat-shock protein (Phillips et al. 1984), we were surprised to find that Lon antigen levels were remarkably constant in cells cultured for 2.5 h at 30°C, 37°C, and 42°C (Fig.4B). Lon levels were also unchanged regardless of *ssrA* or *rnb* genetic background (Fig.4B). We further examined whether Lon levels increase transiently in response to temperature up-shift, but observed no significant increase over the first few minutes of heat stress (Fig.4B, lower blot). These data show that Lon overproduction can activate YoeB, but this mechanism does not account for activation at elevated temperature.

**Figure 5:**
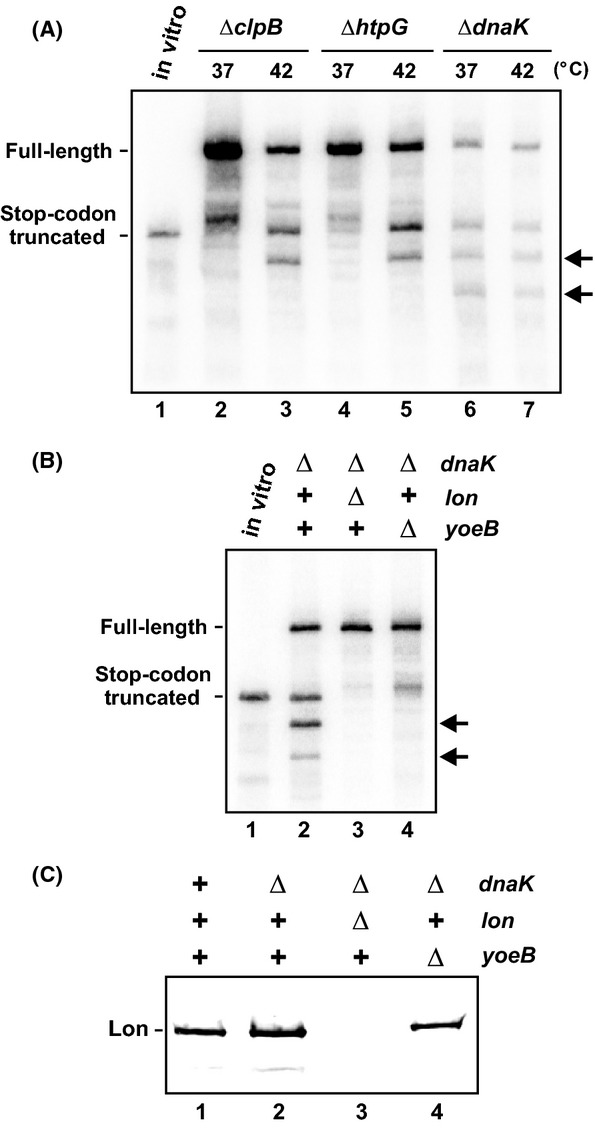
YoeB is activated in Δ*dnaK* mutants. (A) Deletion of *dnaK* induces A-site mRNA cleavage activity. *flag-(m)ybeL-PP* transcripts were expressed in *ssrA*^*−*^ *Δrnb* cells that carry additional deletions in *clpB*, *htpG,* or *dnaK*. The Δ*clpB* and *ΔhtpG* cells were grown at 37°C or 42°C as indicated. The Δ*dnaK* cells were grown at 37°C then maintained at 37°C or shifted to 42°C for the final 30 min of culture. (B) YoeB is activated in Δ*dnaK* mutants. *flag-(m)ybeL-PP* transcripts were expressed in *ssrA*^*−*^ *Δrnb* cells carrying additional gene deletions as indicated. All cells were grown at 37°C and transcripts were detected by northern hybridization. In (A and B), the migration positions full-length and truncated *flag-(m)ybeL-PP* mRNA are indicated. The horizontal arrows indicate additional *yoeB*-dependent truncated transcripts. (C) Immunoblot analysis of Lon in Δ*dnaK* backgrounds. Urea-soluble protein was isolated from *ssrA*^*−*^ *Δrnb* cells carrying additional gene deletions as indicated. All cells were grown at 37°C and Lon antigen was detected using polyclonal antisera.

Heat shock activates the transcription of several genes that facilitate adaptation to thermal stress. We reasoned that an additional heat shock factor may collaborate with Lon to degrade YefM and therefore account for activation at high temperature. To test this hypothesis, we induced the heat-shock regulon through ectopic expression of *rpoH*, which encodes the *σ*^32^ heat-shock transcription factor (Nonaka et al. 2006; Guisbert et al. 2008). Expression of *rpoH* at 37°C induced an mRNase activity that is indistinguishable from that observed in cells grown at 42°C (Fig.4C, lanes 3 and 5). This activity was dependent on *lon* and *yefM-yoeB*, but not *clpP* (Fig.4C, lanes 8, 10, 12, and 14). We tested whether Lon increases in response to *rpoH* expression and found that protease levels remained constant (Fig.4D). Because YoeB is activated at lower temperature through induction of the heat-shock regulon, we tested whether known ATP-dependent chaperones facilitate activation. ClpB, HtpG, and DnaK are all induced during heat shock and have been implicated in the degradation and/or refolding of proteins at high temperature (Sherman and Goldberg 1992; Mogk et al. 1999; Thomas and Baneyx 2000; Huang et al. 2001). Therefore, we examined *flag-(m)ybeL-PP* transcripts expressed in *ssrA*^*−*^ *Δrnb* cells that carry additional deletions of the *clpB, htpG,* or *dnaK* genes. The Δ*clpB* and Δ*htpG* mutations had no effect on transcript processing at either 37°C or 42°C, but the Δ*dnaK* mutant showed increased mRNase activity even at 37°C (Fig.5A). In fact, yet another truncated *flag-(m)ybeL-PP* transcript accumulated in *ssrA*^*−*^ *Δrnb ΔdnaK* cells (Fig.5A, indicated by lowest arrow in lanes 6 and 7). This additional truncation product presumably arises from YoeB-mediated cleavage at an upstream codon within the *flag-(m)ybeL* message. *dnaK* mutations are known to induce the heat-shock regulon at low temperature (Straus et al. 1990), suggesting that YoeB is activated in Δ*dnaK* cells by the same mechanism that underlies toxin activation during ectopic *rpoH* expression. In accord with this model, we found that *lon* and *yefM-yoeB* are both required for increased mRNase activity in the Δ*dnaK* background (Fig.5B, lanes 3 and 4). Additionally, immunoblot analysis showed that Lon protease levels do not increase dramatically in Δ*dnaK* mutants (Fig.5C). Collectively, these results demonstrate that YoeB is activated under three conditions – high temperature, *rpoH* expression and *dnaK* mutation – that induce the heat-shock regulon.

### Thermal activation of YoeB does not inhibit global protein synthesis

**Figure 6:**
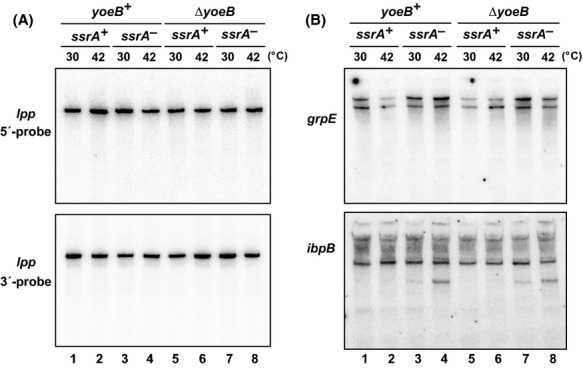
Thermal-induced YoeB activity is not detected on endogenous transcripts. (A) Total RNA from cells grown at 30°C and 42°C was analyzed by northern hybridization using oligonucleotide probes to the 5′- and 3′-untranslated regions of *lpp* mRNA in *yoeB*^*+*^ and Δ*yefM-yoeB* (Δ*yoeB*) backgrounds. (B) The same RNA samples from (A) were analyzed by northern hybridization using probes to the 5′-UTRs of *grpE* and *ibpB* messages.

Previous work has shown that YoeB cleaves *ompA* and *lpp* transcripts in *E. coli* (Winther and Gerdes 2009; Zhang and Inouye 2009). Therefore, we tested whether YoeB cleaves these endogenous mRNAs in response to elevated temperature. We were unable to detect truncated *lpp* transcripts using probes to the 5′- and 3′-untranslated regions (Fig.6A). Similarly, the *ompA* transcript was not cleaved during culture at 42°C (data not shown). We also considered the possibility that YoeB may preferentially cleave heat-shock transcripts and provide a mechanism to fine-tune their translation. However, we did not detect *yoeB-*dependent cleavage in *grpE* and *ibpB* transcripts in response to growth at 42°C (Fig.6B). Although smaller *ibpB* transcripts were detected at 42°C in *ssrA*^*−*^ cells, these fragments accumulated to similar levels in both *yoeB*^*+*^ and Δ*yoeB* backgrounds (Fig.6B). These data are seemingly at odds with the observation that *flag-(m)ybeL-PP* reporter transcripts are efficiently cleaved under the same growth conditions. We hypothesize that YoeB activity is focused on the reporter transcript due to a combination of overexpression and inefficient translation termination. We have shown previously that the entire pool of release factor-1 (RF-1) is sequestered on paused ribosomes during *ybeL-PP* overexpression (Janssen and Hayes 2009). RF-1 depletion then causes other ribosomes to stall at the *ybeL-PP* termination codon with unoccupied A sites (Janssen and Hayes 2009). Because YoeB must compete with translation factors to gain access to its A-site codon substrate, we propose that these latter ribosomes with unoccupied A sites are preferentially targeted by the nuclease.

### Cell growth is not inhibited during YoeB activation

**Figure 7:**
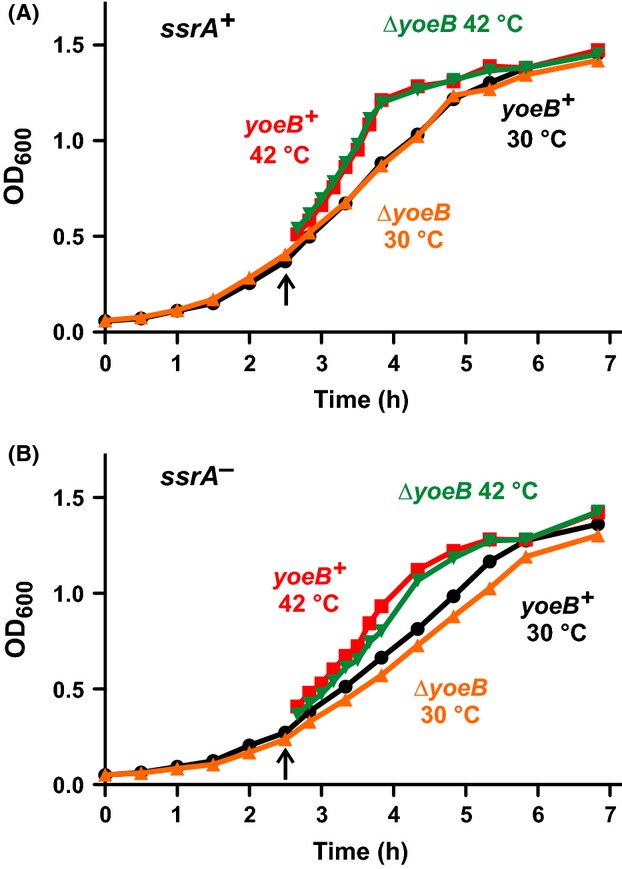
Cell growth is not arrested during heat shock. (A) & (B) E. coli cells with the indicated genotypes were grown in shaking LB broth at 30°C for 2.5 h, then shifted to 42°C (indicated by the upward arrow) for continued culture. Cell growth was monitored by optical density at 600 nm (OD_600_).

**Figure 8:**
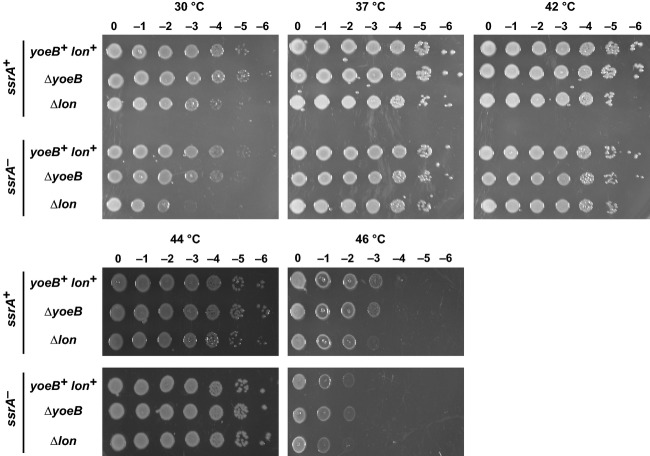
YoeB confers no growth advantage at elevated temperatures. *Escherichia coli* X90 cells of the indicated genetic backgrounds were adjusted to OD_600_ = 1.0, then serial diluted in LB medium and spotted onto LB agar for overnight growth at the indicated temperatures.

The stress-response model of TA function postulates that environmental stress activates mRNase toxins to temporarily inhibit translation while transcription is redirected to stress-response genes (Gerdes et al. 2005). Because YoeB is activated at high temperature, we asked whether acute heat shock leads to temporary growth arrest as predicted by the stress-response model. We monitored the growth of wild-type *yoeB*^*+*^ cells and observed no inhibition of growth during the transition from 30°C to 42°C (Fig.7A). Moreover, Δ*yefM-yoeB* mutants grew along the same trajectory as *yefM-yoeB*^*+*^ cells during the heat-shock treatment (Fig.7A). These results indicate that YoeB activity is not sufficient to arrest growth, consistent with the absence of detectable cleavage within endogenous messages at 42°C. tmRNA facilitates ribosome recycling after YoeB-mediated A-site cleavage, so we also examined the response of *ssrA*^*−*^ cells to heat shock. Although *ssrA*^*−*^ mutants grow more slowly than *ssrA*^*+*^ cells at 30°C and 42°C, the Δ*yefM-yoeB* mutation had little effect in this background (Fig.7B). Because YoeB activity persists over at least 2 h of culture at 42°C, we examined whether the toxin influences cell growth over longer time scales. We spotted *yefM-yoeB*^*+*^ and *ΔyefM-yoeB* cells onto LB agar for overnight incubation at temperatures ranging from 30°C to 46°C, but observed no differences in cell growth in various *ssrA* and *lon* backgrounds (Fig.8). In fact, the most prominent growth defects were observed with *ssrA*^*−*^ cells at 46°C and *Δlon* cells at 30°C. The *ssrA* mutation reduced cell growth at 46°C about 10-fold in the *yefM-yoeB* and *lon* backgrounds (Fig.8). Additionally, Δ*lon* cells grew more slowly than *lon*^*+*^ cells on solid medium at 30°C, and this effect was even more pronounced in the *ssrA*^*−*^ background (Fig.8). Together, these results indicate that tmRNA and Lon contribute to fitness at high and low temperatures, but YefM and YoeB do not confer a discernible advantage under these conditions.

## Discussion

The results presented here reveal temperature-induced mRNase activity in *E. coli* cells. Several observations argue that YoeB is responsible for this nuclease activity. First, transcripts must be actively translated in order to be cleaved. This is in accord with evidence that YoeB binds the ribosome and only cleaves translated messages in vivo (Christensen-Dalsgaard and Gerdes 2008; Feng et al. 2013). Second, temperature-induced mRNase activity requires Lon, consistent with the well-established role for this protease in antitoxin degradation (Gerdes et al. 2005; Tsilibaris et al. 2006; Gerdes and Maisonneuve 2012; Goeders and Van Melderen 2014). Third, expression of *yoeB* at lower temperatures recapitulates heat-induced mRNase activity; and *yefM* overexpression is sufficient to suppress mRNase activity at elevated temperature. Finally, Δ*yefM-yoeB* mutants do not exhibit heat-induced mRNase activity. Collectively, these results indicate that growth at elevated temperatures results in Lon-mediated degradation of YefM and concomitant activation of YoeB. These findings reinforce the conclusions of several previous studies showing that environmental stresses activate TA modules. Gerdes and colleagues have shown that RelE, MazF and other *E. coli* toxins become activated in response to amino acid starvation (Christensen et al. 2001; Christensen and Gerdes 2003). Additionally, the *E. coli dinJ-yafQ* operon contains a LexA box in its promoter, and its transcription is de-repressed in response to DNA damage (Prysak et al. 2009). Thus, our data are broadly consistent with a stress-response function and show that temperature stress is yet another environmental stimulus that activates TA modules.

Although YoeB activity becomes manifest at higher temperatures, the mechanism by which YefM is degraded preferentially over other antitoxins is not clear. Van Melderen and colleagues reported that gratuitous overproduction of Lon is sufficient to activate YoeB, but other known *E. coli* toxins were activated to a much lesser degree (Christensen et al. 2004). Similarly, the data presented here suggest that YoeB is the only toxin activated in response to elevated temperature. Together, these observations indicate that YefM is particularly sensitive to Lon activity. Although Lon is considered a heat-shock protein and *lon* transcription is upregulated during heat shock (Goff et al. 1984; Phillips et al. 1984), we find that Lon levels are essentially the same in cells grown at different temperatures. This argues that increased Lon concentration cannot account for thermal activation. It is also possible that YefM and YoeB dissociate in response to increased temperature, though it appears that YefM-YoeB is no more thermolabile than other TA complexes (Cherny et al. 2005). Moreover, we find that YoeB is activated at 37°C by inducing the heat-shock regulon through ectopic *rpoH* expression. This latter observation raises the possibility that an additional heat-shock factor may collaborate with Lon to degrade YefM. Indeed, there are reports of ATP-dependent chaperones working in concert with Lon to specifically degrade misfolded and damaged proteins (Sherman and Goldberg 1992; Huang et al. 2001). However, the ClpB, HtpG, and DnaK chaperones are not required for the thermal activation of YoeB. In fact, Δ*dnaK* mutants show constitutive activation of YoeB even at low temperature. This finding is consistent with recent data from the Chien and Laub laboratories showing that *Caulobacter crescentus* Δ*dnaK* mutants have increased Lon protease activity (Jonas et al. 2013). They find that unfolded proteins stimulate Lon activity allosterically. Because proteins tend to aggregate and misfold during thermal stress, the findings of Jonas et al. provide an explanation for increased protease activity in the absence of Lon overexpression. Their model may also account for YoeB activation in response to *σ*^32^ overproduction. DnaK binds to *σ*^32^ (Gamer et al. 1992; Liberek et al. 1992), and therefore overproduced transcription factor could potentially sequester the chaperone, preventing it from refolding other client proteins. Thus, super-physiological levels of *σ*^32^ could paradoxically lead to more unfolded proteins and increased Lon activity. Though this model accounts for Lon activity at high temperature, it does not explain why YefM is preferentially degraded while other antitoxins appear resistant to proteolysis. Presumably, YefM carries a unique recognition determinant that allows facile degradation. Given that Lon and YefM/YoeB are distributed widely throughout Gram-negative and Gram-positive bacteria (Nieto et al. 2007; Kumar et al. 2008; Yoshizumi et al. 2009; Sevillano et al. 2012), it will be of interest to determine whether thermal activation is conserved in other species.

Early biochemical studies showed that purified YoeB has intrinsic RNase activity in vitro (Kamada and Hanaoka 2005; Christensen-Dalsgaard and Gerdes 2008); but in vivo, the toxin only cleaves actively translated messages that are bound to the ribosome (Christensen-Dalsgaard and Gerdes 2008; Zhang and Inouye 2009; Feng et al. 2013). YoeB binds to the ribosome A site, where it cleaves the A-site codon to produce a truncated nonstop mRNA (Kamada and Hanaoka 2005; Feng et al. 2013). Although YoeB is generally accepted to be a ribosome-dependent A-site nuclease, there are conflicting reports about which stage of translation is affected. Inouye and colleagues have shown that YoeB acts during translation initiation (Yoshizumi et al. 2009; Zhang and Inouye 2009), whereas Gerdes and colleagues report YoeB-dependent cleavage in termination codons (Winther and Gerdes 2009). Both studies monitored the same endogenous *lpp* and *ompA* transcripts, but the experimental approaches differed. Zhang & Inouye overproduced YoeB from a plasmid vector, whereas the Gerdes group detected endogenous YoeB activity in response to ectopic VapC toxin overexpression (Winther and Gerdes 2009; Zhang and Inouye 2009). Our data show temperature-induced cleavage at two sites, one in the stop codon and another at an unidentified upstream position. Both cleavages are not observed when codon Glu-28 is mutated to an amber stop, indicating that the upstream cleavage site is well downstream of the start codon. Thus, our results are closer to those of Winther et al. and we conclude that endogenous YoeB activated at elevated temperature does not affect translation initiation.

The stress-response model of TA function postulates that activated toxins facilitate changes in gene expression in response to environmental stress (Christensen et al. 2003; Gerdes et al. 2005). The classic example is RelE activation in response to starvation. In this model, amino acid starvation slows protein synthesis, leading to depletion of RelB antitoxin due to its intrinsic instability. RelE then acts as an A-site nuclease to cleave translated messages and inhibit translation. The resulting stalled ribosomes are recycled by tmRNA-SmpB, which provides a burst of amino acids through proteolysis of ssrA-tagged nascent chains (Christensen and Gerdes 2003). The starved cells then alter transcription to express genes for amino acid biosynthesis and a new steady-state is eventually achieved. In principle, YoeB could function in the same manner to accelerate mRNA decay so that new heat-shock transcripts are translated more rapidly. However, we were unable to detect YoeB-dependent cleavages within endogenous messages. YoeB must compete with translation factors for access to the ribosome A site, and therefore it is more likely to cleave mRNA in the context of arrested ribosomes. Because heat shock does not inhibit global translation, YoeB activity is presumably limited to a small population of ribosomes that encounter difficulties during protein synthesis. In contrast, amino acid starvation stress not only activates RelE toxin, but also blocks protein synthesis to produce stalled ribosome complexes for RelE-mediated mRNA cleavage. Another discrepancy between our results and the stress-response model is the absence of growth arrest upon YoeB activation. In fact, *E. coli* cells show no growth lag in response to temperature up-shift, but rather accelerate their growth rate immediately. Moreover, YoeB activity is detectable over several hours of growth at elevated temperature. These data demonstrate that toxins can be activated at low levels, suggesting that they may actually promote more efficient protein synthesis under stress conditions. Heat shock and temperature stress has been shown to damage translation complexes and disrupt protein synthesis (Korber et al. 2000; Jiang et al. 2009). In our model, YoeB activity would perform a quality control function to help recycle translation complexes that stall stochastically during thermal stress. This is still adaptation to environmental stress, yet the proposed function is distinct from previously established roles for toxins in growth arrest and persistence (Maisonneuve et al. 2011; Gerdes and Maisonneuve 2012; Maisonneuve and Gerdes 2014).
